# Intravitreal vascular endothelial growth factor inhibitor systemic and renal toxicity registry

**DOI:** 10.1093/ckj/sfaf206

**Published:** 2025-06-27

**Authors:** Matthew D Nguyen, Ryan Fekrat, Caroline Gee, Arif Nihat Demirci, Sohrab Kharabaf, Dao Le, Mina Tadros, Vu Q Nguyen, Samir Patel, Tai Truong, Rebecca Ahdoot, Ira B Kurtz, Michael Kerr, Abanoub Massoud, Ramy Hanna

**Affiliations:** University of California Irvine, School of Medicine; University of California Irvine, Department of Medicine; University of California Irvine, School of Medicine; Mugla Sitki Kocman University, Faculty of Medicine; University of California Irvine, School of Medicine; University of California Irvine, Department of Medicine; University of California Los Angeles, Department of Biological Sciences; University of California Irvine, Department of Medicine; University of California Irvine, Department of Medicine, Division of Nephrology, Hypertension, and Renal Transplant; University of California Irvine, Department of Medicine, Division of Nephrology, Hypertension, and Renal Transplant; University of California Irvine, Department of Medicine, Division of Nephrology, Hypertension, and Renal Transplant; University of California Los Angeles, Division of Nephrology; Reaction Gears Electronic Media Company; AM consulting company; AM consulting company; University of California Irvine, Department of Medicine, Division of Nephrology, Hypertension, and Renal Transplant

**Keywords:** IVEGFi, nephrotic syndrome, nephrotoxicity, registry, thrombotic microangiopathy

## Abstract

**Background:**

Intravitreal vascular endothelial growth factor inhibitors (IVEGFi) are used in the treatment of diabetic retinopathy, age-related macular degeneration (AMD) and central retinal vein obstruction. As we have previously reported, there are an increasing number of cases documenting IVEGFi with renal injury and increased concentrations in the serum. To assess this claim, we have developed a novel reporting system through an electronic registry for cases of suspected VEGFi injury.

**Methods:**

A website with multiple data protection sets was created to educate, promote awareness and capture patient cases of suspected IVEGFi toxicity. The website displays the molecular biology of VEGF signaling, the process of absorption into the bloodstream, and study reports showing risks on case, cohort and epidemiologic levels. A Health Insurance Portability and Accountability Act (HIPAA)-compliant patient intake form was designed to collect renal, cardiovascular, cerebrovascular, renal biopsy and function data along with drug type, indication and frequency of administration.

**Results:**

In our updated cohort we added 16 total cases from the literature showing signs of renal injury from the patient population receiving VEGFi. In current literature, 46 cases of VEGFi-related renal injury have been documented. To them, we add our 16 cases for a total of 62 cases.

**Conclusion:**

The current database for VEGFi-related nephrotoxicity constitutes the largest case series presented for this condition. This study opens the door for future studies to evaluate what subgroups experience acute kidney injury, proteinuria and hypertension exacerbations. Additionally, we may expand on our database to include timeline markers for symptomatic-correlative VEGFi usage and, in time, predictive measures on a larger scale to correlate comorbidity/drug use with drug effect and mechanism of action.

KEY LEARNING POINTS **What was known:**

- Intravitreal vascular endothelial growth factor inhibitors (VEGFi) have been commonly used to treat various ocular diseases; however, there has been a paucity in the literature of their systemic effects, particularly towards renal function.

**This study adds:**

- Our study adds 16 new cases of adverse renal effects associated this medication.

**Potential impact:**

- This study emphasizes the need and importance for further clinical studies to investigate the renal and systemic risks of intravitreal VEGFi.

## INTRODUCTION

Vascular endothelial growth factor inhibitors (VEGFi) are potent inhibitors of angiogenesis used in solid organ malignancies, and are regarded as chemotherapeutic agents [1]. They consist of monoclonal antibodies or fusion proteins that inhibit activation of VEGF receptors, thereby acting as powerful antiangiogenesis tools [2, 3]. These medications, when given systemically, have proven to be efficacious for retinal pathologies, but there have been adverse observations by some investigators including hypertension (HTN), proteinuria and renal injury, and with some biopsy-proven cases showing thrombotic microangiopathy, endotheliosis, nephrotic syndrome and renal injury [4] (Table 1). This is now a complication of their use well-known to nephrologists, oncologists and onco-nephrologists.

**Table 1:** Cases reported to our IVEGF systemic and renal toxicity database from 2020 to 2024.

Reference	*n*	Agent used	Clinical effect(s), renal pathology
Hanna *et al.* [12]	3	Bev (Cases 1, 2), Aflib (Case 3)	Case 1 and Case 2: DN and chronic TMA (Biopsy+); Case 3: FSGS with chronic TMA features (Biopsy+)
Hanna *et al.* [4]	1	Bev→Ran	Worsening HTN and proteinuria, lessened with Ran use vs Bev
Hanna *et al*. [47]	4	Bev + Ran	Case 1: *de novo* MCD (Biopsy+); Cases 2–4: increased proteinuria, CKD progression, HTN worsening
Phadke *et al.* [5]	1	Ran→Aflib	(Biopsy +) CFSGS + chronic TMA, low serum VEGF level; worsening renal disease and HTN with switch from low potency agent (Ran) to high potency agent (Aflib)
Shye *et al.* [1]	3	Case 1 Bev→Ran, Case 2 Bev, Case 3 Bev→Ran	All: increased proteinuria, CKD progression, HD; Case 1: worsening proteinuria, CKD progression, HD (Biopsy+); Case 2: DN + FSGS with collapsing features + AIN (Biopsy+); Case 3: DN + AIN + low systemic VEGF level
Diabetic Retinopathy Clinical Research Network [48]	3	Bev	Decreased eGFR
Cheungpasitporn *et al.* [49]	2	Bev	Case 1, MGN; Case 2 TMA (Biopsy+)
Georgalas *et al.* [50].	2	Ran + Bev	Decreased eGFR; HD started
Hanna *et al.* [51]	1	Bev	Case 1: scleroderma renal crisis and TMA induced after IVEGFi and oral corticosteroids
Jamrozy-Witkowska *et al.* [52]	1	NR	Decreased eGFR
Kenworthy *et al.* [53]	1	Bev	Increased proteinuria
Khneizer *et al.* [54]	1	Bev	MGN (Biopsy+)
Yoshimoto *et al.* [55]	1	Aflib	Case 1: hypertensive hemorrhage with undetectable VEGF plasma levels after intravitreal injection (preprint)
Morales *et al.* [56]	1	Ran	DN (Biopsy+)
Nobakht *et al.* [57]	1	Bev→Ran→Aflib	CFSGS (Biopsy+) + low systemic VEGF level
Pellé *et al.* [58]	1	Ran	TMA (Biopsy+)
Pérez-Valdivia *et al.* [59]	1	Bev	Relapsed MCD (Biopsy+)
Sato *et al.* [60]	1	Bev	Relapsed MCD (Biopsy+)
Touzani *et al.* [61]	1	Bev	Endotheliosis/possible TMA (Biopsy+)
Tran *et al.* [62]	1	Bev	AIN (Biopsy+)
Yen *et al.* [63]	1	Bev	TMA (Biopsy+)
Gan *et al.* [64]	1		MPGN post-VEGFi (intravitreal)
Anto *et al.* [65]	1		Case report MGN post-VEGFi (intravitreal)
Crowe *et al.* [66]	1		Worsening HTN, drop in eGFR, worsening proteinuria No renal biopsy
Zhang *et al.* [67]	1		TMA and ATN post-IVEGFi
Lou *et al.* [68]	9		Series of 9 patients showing worsening TMA, HTN, proteinuria and AKI/CKD in patients with DR treated with VEGFi (intravitreal)
Ahmed *et al.* [69]	1		Clear worsening AKI and renal function after VEGFi, but no e/o TMA or other NS on biopsy
Nguyen *et al.* (2025) (under review)	16	Various	16 patients recently added to IVEGFi toxicity registry
Total	62		

Biopsy only if “(Biopsy+)” stated.

Data from Shye *et al. Clin Kidney J* 2020 and Phadke *et al. Clin Kidney J* 2021. Adapted under open access license and with proper attribution.

Aflib, aflibercept; AIN, acute interstitial nephritis; Bev, bevacizumab; Biopsy+, biopsy obtained; CFSGS, collapsing focal and segmental sclerosis; DN, diabetic nephropathy; e/o, evidence of; FSGS, focal segmental glomerular sclerosis; HD, hemodialysis started; MCD, minimal change disease; MGN, membranous glomerulonephritis; *n*, number of patients; NR, not recorded; NS, nephrtic syndrome; Ran, ranibizumab; TMA, thrombotic microangiopathy.

**Figure 1:**
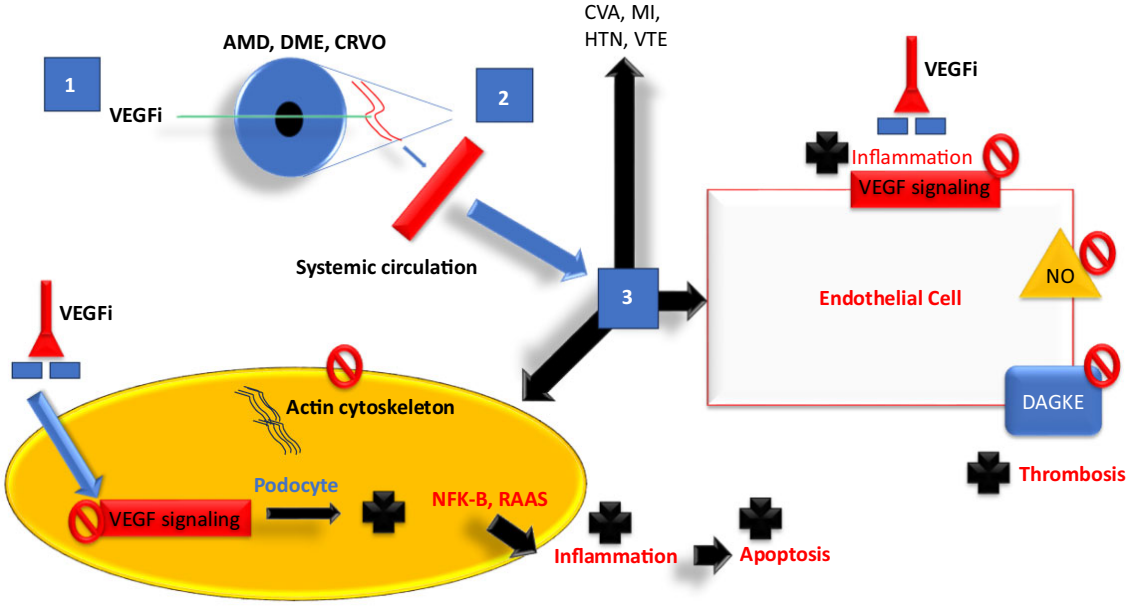
Basic scientific evidence showing cellular changes, transcription changes, biomarker perturbations and renal injury in animal models is not lacking, and its half-life, absorption and proposed mechanism, as well as effects on systemic, coagulation and podocyte, and endothelial effects of IVEGFi. CRVO, central retinal vein occlusion; VTE, venous thromboembolism; NO, nitric oxide; DAGKE, Diacylglycerol Kappa Epsilon; NFK-B, nuclear factor-kappa B; RAAS, renin–angiotensin–aldosterone system.

The basic scientific evidence showing cellular changes, transcription changes, biomarker perturbations and renal injury in animal models is not lacking, and its half-life, absorption and proposed mechanism, as well as effects on systemic, coagulation and podocyte, and endothelial effects of intravitreal VEGFi (IVEGFi) are presented in Fig. 1. There have been mixed data in prospective and retrospective studies documenting our hypothesis that patients who receive IVEGFi for long periods of time, at high doses or who have higher rates of absorption may be at risk for adverse individual medical events and population level complications.

Given the various complexities of drug structure, weight, absorption, dosing and comorbidities, more rigorous controlled studies, and advanced biomarker data are needed from a well-designed trial. Until such data are available, our group at the University of California Irvine (UCI) sought to create an online registry to report suspected cases of IVEGFi renal and systemic toxicity as a pilot project to obtain data to support the increasingly plausible hypothesis that these agents may predispose to worse renal and systemic outcomes particularly in vulnerable patients, or patients who are exposed to high doses of these medications intravitreally. To the literature we add a total of 16 new cases documenting a relationship between VEGFi and renal toxity from our registry (Table 2).

**Table 2:** Registry website reports 2025.

Age (years)	Gender	Comorbidities	Reason for the intravitreal drug	Drug frequency	Drug	Effects reported for drugs
30	Male	CKD, diabetes, HTN	DME	Every 2 weeks	Bevacizumab	sCr 1.4→2→3 since starting avastin 2022→2024. UPCR 6 g. MACR 1.3 g→4 g 2022→2024. HTN worsening. +DR DM/DN
36	Male	CKD, diabetes, HTN	DME	Monthly	Bevacizumab	sCr 1.3→1.5→2.5 over last 2 years. Proteinuria 1→2.7→3.5 g over last 2 years
65	Female	CKD, diabetes, HTN	DME	Every 2 weeks	Bevacizumab	Avastin likely drug. sCr increase 0.7 to 1.2
62	Male	CKD, diabetes, dialysis, heart attack, HTN	Every 3 months new VEGFi (q 3 months—faricimab)	Bi-monthly or less often	Other	Proteinuria in allograft of transplant patient 1→ 4.3 g over last period post 1st injection. Allograft biopsy planned. 1.3 sCr→1.5 mg/dL
61	Male	CKD, diabetes, heart attack, HTN	DME	Monthly	Bevacizumab	sCr worsening from 1.9–2 up to 3.7 mg/dL and rua showing 500+ proteinuria (nephrotic range); no MACR or UPCR
53	Female	CKD	DR	Monthly	Bevacizumab	From time of initiation sCr increased to 7–10 g proteinuria. Rapid progression of CKD
47	Female	CKD, diabetes, heart attack, HTN	Diabetes	Monthly	Ranibizumab	After intravitreal ranibizumab injection, existing proteinuria raised to a nephrotic level in multiple occurrences. Renal biopsy performed when proteinuria was 14 g/day and serum albumin 2.9 g/dL. It showed DN and acute TIN (eosinophil dominant inflammation and tubulitis). The patient has Class 3 obesity, as well
79	Male	CKD, diabetes	AMD	Monthly	Aflibercept	New-onset proteinuria from 1+ to 3+. 140 mg/day uACR to 1 g/day
47	Male	Anemia, CKD, diabetes, HTN	Diabetic retinopathy got 6 weeks before injury per pt (last known well kidney function 1.5→3.6)	Every 2 weeks	Bevacizumab	Nephrotic-range proteinuria 10 g of protein. Biopsy suggested
43	Female	CKD, HTN	VEGFi for DME	Monthly	Bevacizumab	200 mg/g of proteinuria (albuminuria) initially in 2017 when started IVEGF→increase to 10 g/day of proteinuria
67	Female	CKD, diabetes, heart attack, HTN	DME	Monthly	Bevacizumab	Patient with worsening renal function (accelerated DM and DN)
75	Male	HTN	AMD	bi-monthly or less often	Aflibercept	Worsening HTN, no proteinuria or hematuria
74	Male	CKD, HTN	Macular degeneration	Monthly	Aflibercept	No proteinuria noted, but accelerated HTN, with swings. HTN diagnosis seemed to start around time of starting IVEGFi
53	Male	CKD, diabetes, HTN	DR	Bi-monthly or less often	Bevacizumab	Nephrotic-range proteinuria
58	Male	CKD	DM	Bi-monthly or less often	Bevacizumab	MACR went from 1.3→5 g in span of 5 months. UPCR 4.7 g
47	Female	HTN	Proliferative diabetic retinopathy	Monthly	Other	Worsening HTN and worsening proteinuria

sCr, serum creatinine; MACR, microalbumin creatinine ratio; DN, diabetic nephropathy; rua, random urine analysis; TIN, tubulointerstitial nephritis; uACR, urine albumin creatinine ratio.

## MATERIALS AND METHODS

We obtained a UCI institutional review board (IRB) approval, UCI IRB protocol #2472. Using funds made generously available by the UCI Chairman's grant in 2021–22, a robust website with multiple data protection sets was organized to educate, to allow for compiling of publications from our group on the topic to promote awareness and to capture patient cases of suspected IVEGFi toxicity. The website focused on displaying the molecular biology of VEGF signaling and pathophysiology induced by VEGF depleting monoclonal antibodies (mAb) and ligand binding synthetic antibodies, the process of absorption of intravitreal VEGF mAb into blood stream and evidence that this occurs to a clinically significant degree, the reporting of cases, and reporting of studies showing risks on case, cohort and epidemiologic/population levels. A Health Insurance Portability and Accountability Act (HIPAA)-compliant patient intake form was designed to collect renal, cardiovascular, cerebrovascular, renal biopsy and renal function, filtration data, drug type, indication and frequency of administration. The website was provided by contracting group Reaction Gears Electronic Media Company.

We also used social media (especially X, formerly Twitter) as the main platform to disseminate information and recruit patients in a wide-reaching and cost-effective manner. From 2021 to 2024 data were collected from physicians reporting these side effects and patients regarding side effects. The total number of IVEGFi injections is nearly 6 million, in 2 million patients. This documents how common these reactions are, as well as the level of awareness of these side effects amongst the nephrological, primary care and ophthalmological community. We collected the data in the UCI-supported IVEGF inhibitor registry from 2021 to 2024, which we now are presenting (website: https://intravitreal-vegf-inhibitor-nephrotoxicity-registry.org).

## RESULTS

There were 16 additional reported cases that were added to our IVEGFi database registry. Of note all data have been reported and all data provided were subjcted to the input of those who have reported.

The first was a 30-year-old male with chronic kidney disease (CKD), diabetes mellitus (DM) and diabetic retinopathy (DR), receiving every 2 weeks bevacizumab, who experienced subacute acute kidney injury (AKI) with a rise of serum creatinine (sCr) from 1.4 to 3 mg/dL from 2022 to 2024, with an increase in proteinuria from sub-nephrotic (1.3 g) to 4 g. This was accompanied by accelerated HTN in addition to the accelerated CKD progression. The second case was a 36-year-old male with CKD, DM, HTN and DR, who experienced a near doubling of sCr from 1.3 to 2.5 mg/dL along with a rise of proteinuria from 1 to 2.5 g over a 2-year time span after starting monthly bevacizumab. The third case was a 65-year-old female with CKD, DM, DR and HTN, with a rise in sCr from 0.7 to 1.2 over the year after starting every 2 weeks bevacizumab. The fourth case was a 62-year-old male with CKD, DM, myocardial infarction (MI) and HTN, who was on dialysis but received a transplant. Post-transplant he was started on every 3 months faricimab, and the patient noted an increase of proteinuria from 1 to 4.3 g, with a rise of sCr from 1.3 to 1.5 mg/dL. Subsequent workup did not show evidence of rejection.

The fifth case was a 61-year-old male with CKD, MI, HTN, DM and DR, with sCr worsening from 1.9 to 3.7 and nephrotic-range proteinuria after receiving monthly bevacizumab. The sixth case was a 53-year-old female with CKD, DM and DR, with accelerated CKD progression and proteinuria increase to nephrotic-range proteinuria with 10 g of protein/day after monthly bevacizumab. The seventh case was a 47-year-old female with CKD, type 2 DM, MI and HTN, on monthly ranibizumab; the patient underwent accelerated CKD, and a rise from sub-nephrotic to 14 g/day proteinuria. The patient underwent a renal biopsy showing tubulointerstitial nephritis and diabetic nephropathy. The eighth case was a 79-year-old male with CKD, DM and AMD receiving monthly aflibercept with new-onset proteinuria that worsened from macroalbuminuria to 1 g/day of proteinuria.

The ninth case was a 47-year-old male with anemia, CKD, DM, HTN and DR, with rapid rise of sCr from 1.5 to 3.6 mg/dL over 6 weeks after starting every 2 weeks bevacizumab. Additionally, the patient developed 10 g of proteinuria; a biopsy was suggested but no results were reported. The tenth case was a 43-year-old female with CKD, HTN, DM and DR/diabetic macular edema (DME) receiving monthly bevacizumab with increasing proteinuria from 200 mg/day to 10 g per day.

The 11th case wa a 67-year-old female with CKD, DM, DR, MI and HTN receiving monthly bevacizumab with accelerated CKD progression reported.

The 12th case is a 75-year-old male with HTN and AMD receiving aflibercept bimonthly or less, who developed worsening HTN but no proteinuria or hematuria. The 13th case is a 74-year-old male with CKD, HTN and AMD receiving monthly aflibercept who developed accelerated hypertensive paroxysms coinciding with VEGFi injections. The 14th case was a 53-year-old male with CKD, DM, HTN and DR receiving bimonthly or less bevacizumab who develop worsening of proteinuria to nephrotic range. The 15th case was a 58-year-old male with CKD, DM and DR receiving bimonthly or less often bevacizumab, who had his microalbumin to creatinine ratio increase from 1.3 to 4.7 g/day (nephrotic range) in the span of 5 months after starting injections.

The 16th and final patient was a 47-year-old female with HTN, DM and DR (proliferative diabetic retinopathy lesion provided) who was receiving an undisclosed VEGFi at an undisclosed frequency, who was noted to have worsening HTN and proteinuria after injections started.

The average age of our cases was 56.4 years old, there were 10 males and 6 females. The indications for VEGFi use was DR/DME in 13 patients, and AMD in 3 patients. The drug used most often was bevacizumab in 10 patients, aflibercept in 3 patients, faricimab in 1 patient, ranibizumab in 1 patient, and unreported in 1 patient (see Table 2).

## DISCUSSION

This case series adds a total of 16 cases to the current literature as part of our registry. IVEGFi were previously thought to avoid significant systemic absorption, with levels reaching <200-fold systemic level injections [5], [6]. However, recent pharmacokinetic studies by Avery *et al*. in 2014 and 2017 showed that IVEGF blockade results in serum concentrations approximating or exceeding 50% inhibitor concentrations (IC50) [7–9]. The current IVEGFi agents in use display varying systemic potencies, with bevacizumab showing the greatest systemic exposure, followed by aflibercept and then ranibizumab [7–9].

There has been, admittedly, mixed data in prospective and retrospective studies. Randomized controlled trials focused on investigator reported side effects. We postulate that given the lack of involvement of nephrologists and lack of screening for proteinuria as well as renal injury markers, it is possible to understand the lack of consistently positive reports as limitations of patient selection and testing. Table 3 shows studies that have showed a different clinical outcome (and the level of association), seen between IVEGFi and various important clinical and biomarker outcomes.

**Table 3:** Positive studies showing effect between IVEGFi and renal function.


**(A) Pharmacological studies**		
Absorption in AMD, dec. systemic VEGF (Bev, Aflb) > Ran	Prospective observational study	Avery
Absorption in AMD/DME/CRVO, dec. systemic VEGF (Bev, Aflib)>Ran	Prospective observational study	Avery
Dec. systemic VEGF (Bev, Aflib) > Ran	Prospective randomized clinical study	Jampol
Absorption of drug in AMD, dec. systemic VEGF	Retrospective study of RCT data	Rogers
Dec. systemic VEGF (Bev, Aflib)	Prospective randomized observational study	Zehetner
Bev > Ran dec. in systemic VEGF	Prospective observational study	Yoon
Dec. systemic VEGF (Bev, Aflib)	Prospective non randomized clinical study	Hirano
**(B) Animal studies/biomarker studies**		
Absorption of drug, binding at glomerulus	Animal (simian) study	Tschulakow
NGAL, KIM-1, HIF-1alpha, Nephrin levels increase post VEGFi (intravitreal injection) suggesting renal tissue injury	Basic science study	Chebotareva
**(C) Clinical studies showing changes in blood pressure**		
Higher blood pressure linked to need for more VEGFi	Retrospective study	Shah
Limited short-term rise in blood pressure at 1 h	Prospective observational study	Lee
Long- and short-term rise in systolic blood pressure	Observational study	Rasier
**(D) Clinical studies showing changes in proteinuria**		
Increased proteinuria 45% of patients (not statistically significant)	Prospective observational study	Bagheri
Significant rise in diastolic blood pressure		
Significant rise in hemoglobin and platelets		
4% of patients with AKI and elevated UPCR after VEGFi	Retrospective observational study	Jalalonmuhali
Significant rise in UPCR in patients with preexisting proteinuria	Prospective observational study	Chung
**(E) Studies showing changes in renal function**		
Increase risk of AKI in male patients and those with eGFR >30 mL/min	Retrospective cohort study	Bunge
Increase in MACR and drop in eGFR post-intravitreal injection	Retrospective cohort study	Del Cura Mar
Injected cohort with greater drop in eGFR than controls	Retrospective cohort study	Ou
Injected cohort of diabetic patients demonstrates greater drop in eGFR than in controls	Retrospective observational cohort study	Rivero
85%–120% relative risk increase of dialysis need in VEGFi (intravitreal) treated individuals	Retrospective cohort study using national database	Yang
33% increase risk of CKD progression in patients treated with VEGFi (intravitreal) compared with laser photocoagulation	Retrospective cohort study	Chen
18 cases showing worsening HTN, eGFR and UPCR/MACR	Registry of clinical events	Hanna *et al.* (unpublished)
Elevated risk but similar risk of renal injury amongst VEGF inhibitors (Bev = Ran = Aflib = Faric)	Meta-analysis	Cai
**(F) Epidemiological studies showing increase in population adverse events**		
Increased risk of CVA in DME patients	Meta-analysis	Avery
Increased all-cause mortality in AMD patients	Retrospective observational study	Hanhart
Increased risk of mortality after MI in AMD patients	Retrospective observational study	Hanhart
Increased risk of mortality after CVA in AMD patients	Retrospective observational study	Hanhart
Increased risk of thrombotic events	Clinical trial database retrospective	Schmid
Increased risk of death in DM patients treated with VEGF blockade	Meta-analysis	Lees

Aflib, aflibercept; Bev, bevacizumab; CRVO, central retinal vein obstruction; dec., decreased; Faric, faricimab; HIF 1alpha, hypoxia inducing factor 1 alpha; KIM-1, kidney injury molecule-1; MACR, microalbumin to creatinine ratio; *n*, number of study subjects; NGAL, neutrophil gelatinase associated lipocalin; Ran, ranibizumab; RCT, randomized controlled trial; SAE, serious adverse event.

There have been 32 case reports detailing systemic effects of IVEGFi, describing worsening HTN, *de novo* or worsening proteinuria, thrombotic microangiopathy, collapsing focal and segmental sclerosis, and minimal change disease [1, 2, 5]. This includes multiple case studies reported by our group, including biopsy findings that reinforce TMA as a pathognomonic lesion of VEGF blockade, with associated collapsing focal segmental glomerulosclerosis [5].

However, other systematic studies of IVEGFi have given mixed results, with some studies demonstrating negative associations between IVEGFi and worsening HTN and proteinuria. For instance, Glassman *et al*. and Kameda *et al.* did not report significant negative effects of VEGFi on blood pressure, proteinuria or renal function in larger-scale studies [10, 11]. Confounding factors may include differential vitreous absorption, total drug dose, genetics and comorbidities [12]. Chung *et al.* found worsening urine protein creatinine ratio (UPCR) only in patients already near nephrotic-range proteinuria, suggesting that VEGFi may preferentially affect patients with pre-existing diabetic nephropathy [13]. Thus, there may be an unidentified patient subgroup at greater risk of systemic and renal toxicity from IVEGFi. The varied effects of VEGFi in the literature have demonstrated the need for systemic documentation of all possible side effects of these agents and increased pharmacovigilance of their possible renal toxicity.

As this effect has been more closely scrutinized, more data have come from various locations supporting the hypothesis that patients who receive IVEGFi for long periods of time, at high doses or who have higher rates of absorption may be at risk for adverse individual medical events and population-level complications (Table 3). Most concerning, Yang *et al.* showed a near doubling of end-stage renal disease relative risk in patients receiving IVEGFi [14]. There are, however, other studies that show no statistically significant effects between IVEGFi treated and control arms [15]. There are other trials that did not show a significant effect between IVEGFi treated and control arms, for a balanced perspective (Table 4).

**Table 4:** studies not showing effect despite IVEGFi.

Systemic effect/pathology	Study type	Study name/reference
**(A) Trial data analysis**		
No increase in AE reporting	Post-hoc trial analysis	Jiang
**(B) Effects on HTN after intravitreal injection**		
No significant change in blood pressure	Observational study	Risimic
**(C) Renal and proteinuria studies**		
No association of IVEGFi and AKI	Meta-analysis	Tsao
No change in eGFR 7–30 days after injection (Bev, Aflib, Ran)	Retrospective observational study	Kameda
No long-term change in HTN or category of albuminuria	Planned retrospective analysis of trial	Glassman
No association with # VEGFi injections and proteinuria	Retrospective observational study	O’Neill
Significant rise in UPCR in patients without preexisting proteinuria	Prospective observational study	Chung
**(D) Population studies showing increased morbidity and mortality**		
No difference in AE between Bev, Ran, Faric, Aflib and sham	Random effects meta-analysis	Jhaveri
No finding of CVA, MI, all-cause mortality in AMD patients	Retrospective observational study	Dalvin
No finding of increased CVA in DME patients	Retrospective observational study	Starr
Ran versus Bev same number of reported SAE	Clinical trial database retrospective	Ran/Bev Trial N

AE, adverse events; Aflib, aflibercept (Eylea®); Bev, bevacizumab (Avastin®); Faric, faricimab (Vabysmo ®); Ran, ranibizumab (Lucentis®); Ran/Bev Trial N, Ranibizumab Bevacizumab Trial Network; SAE, severe adverse event.

There is a theoretical difference between half-life, molecular weight and absorption, which have shown a predilection for certain agents to be absorbed more heavily than others. While other trials, like the important publication Cai *et al.*, showed similar systemic effects between the different pharmacological agents (bevacizumab, aflibercept, ranibizumab and faricimab) when used intravitreally [16]. Even more complexity is added when the observation is noted that patients who tend to have worse renal and vascular complications seem to be preferentially at risk for adverse outcomes, meaning risk may vary widely between patients.

### Update: positive studies (Table 3)

A number of positive studies were obtained showing absorption of IVEGFi in human subjects, starting with the work of Avery *et al.* in 2014 and 2017. These studies showed significantly elevated VEGFi concentrations when given intravitreally for prolonged periods of time above the IC50 [8, 9]. Jampol, Roger, Zehetner, Hirano and Yoon all confirmed VEGFi absorption and depletion of systemic VEGF [17–21]. Animal studies by Tschulakow *et al.* showed binding of VEGFi in simian glomeruli 1 week after intravitreal injection [22]. Chebotareva *et al.* showed biomarkers in renal tissue indicating renal injury post-IVEGFi injection [23].

Clinical studies showing increase in blood pressure include Shah *et al.*, Lee *et al*. and Rasier *et al.* [24–26]. Proteinuria was shown to be elevated post-VEGFi injections intravitreally in Bagheri *et al.*, Jalalonmuhali *et al.* and Chung *et al.* showed increases in proteinuria preferentially in patients with already elevated levels of proteinuria (A3) [13, 27, 28].

Bunge *et al.* showed increases in AKI risk in certain subgroups post-VEGFi intravitreal injection in male patients and those with estimated glomerular filtration rate (eGFR) >30 mL/min [29]. Del Cura Mar *et al.* showed increased risk of rising microalbumin to creatinine ratio (MACR) and eGFR drop post-VEGFi intravitreal injection [30]. Yang *et al.* showed 85%–120% increased relative risk of end-stage renal disease/dialysis dependence in VEGFi intravitreal injection–treated populations [14]. Chen *et al.* showed 33% increased risk of CKD progression with IVEGFi versus those who got photocoagulation [31]. Ou *et al.* and Rivero *et al.* noted a drop in eGFR in their injected cohort of diabetic patients compared with their control [32, 33]. Cai *et al.* showed the elevated risk of renal injury amongst all patients treated with bevacizumab, ranibizumab, aflibercept and faricimab, with no one agent being safer than the other [16].

Epidemiological studies by Hanhart *et al.* showed increased all-cause mortality, mortality after myocardial infarction, mortality after cerebrovascular accident in AMD patients treated with IVEGFi agents [34–36]. Avery *et al.* showed increased risk of cerebrovascular accident (CVA) in DME patients [37]. Schmid *et al.* [38] showed an increased risk of thrombotic events in VEGFi-treated patients, Lees *et al.* [39] showed increased risk of death in DM with DME patients treated with IVEGFi.

### Update: negative studies (Table 4)

In order to fairly balance the body of evidence being produced we address that there have been a number of studies showing no differences between patients receiving VEGF injections and matched controls. Jiang *et al.* showed no change in rate of adverse event reporting from pharmacovigilance [40]. Risimic *et al.* in an early studied showed no difference in blood pressure post IVEGFi [41]. Tsao, Kameda, Glassman, O'Neill and Chung *et al*., while showing some subgroup differences, showed no significant difference for patients who had no pre-existing proteinuria [10, 11, 13, 42, 43]. A random effect meta-analysis showed no difference between different drug types causing renal function deterioration between three different intravitreal injections and sham injections. Dalvin *et al.* in a retrospective observational trial showed no difference in cerebrovascular, cardiovascular or all-cause mortality in AMD patients treated with IVEGFi versus controls [44]. Starr *et al.* showed no finding of increased CVAs in DME IVEGFi-treated patients vs controls [45]. Analysis of the ranibizumab/bevacizumab trial clinical data base analysis showed no difference in serious adverse events between ranibizumab and bevacizumab [46].

## CONCLUSION

We add to the literature 16 reports of cases noting increasing HTN, accelerated CKD progression and worsening proteinuria, 1 report of interstitial nephritis with accelerated CKD progression, and nephrotic syndrome post-initiation or re-initiation of IVEGFi. We review the literature as above to show the heterogenous body of data showing studies without a significant effect of IVEGFi on renal parameters and epidemiological outcomes. We contrast that with the increasing body of data, including large national database studies, showing increased risk of CKD progression, proteinuria worsening, end-stage renal disease/dialysis dependence, along with increased all-cause mortality, and mortality post-CVA and -MI. At this time our group still maintains this registry and welcomes any and all reports of this being observed clinically, or suspected clinically. The aim is to eventually design well-controlled studies that target those patients most likely to experience negative renal and systemic effects (comorbid HTN, proteinuria and CKD). These studies can help confirm whether Cai *et al.*’s retrospective observations showing that all agents are equally risky, or whether ranibizumab or faricimab have differential toxicity. The use of biomarkers in any study can also help catch short term renal injury not easily quantifiable over the short term as suggested by Chebotareva *et al.* in animal models. A non-industry-based, independent, National Institutes of Health trial is urgently needed to address the growing and alarming body of literature on the renal and systemic risk factors of IVEGFi, and reports of worsened renal, cardiovascular, systemic and epidemiological outcomes.

## Data Availability

The data underlying this article are available in the article and in its online supplementary material.
